# Contribution of Dietary Oxalate and Oxalate Precursors to Urinary Oxalate Excretion

**DOI:** 10.3390/nu13010062

**Published:** 2020-12-28

**Authors:** Joseph J. Crivelli, Tanecia Mitchell, John Knight, Kyle D. Wood, Dean G. Assimos, Ross P. Holmes, Sonia Fargue

**Affiliations:** Department of Urology, University of Alabama at Birmingham School of Medicine, FOT 1120, 1720 2nd Ave S, Birmingham, AL 35294-3411, USA; crivelli@uab.edu (J.J.C.); taneciamitchell@uabmc.edu (T.M.); johnknight@uabmc.edu (J.K.); kylewood@uabmc.edu (K.D.W.); deanassimos@uabmc.edu (D.G.A.); sfargue@uab.edu (S.F.)

**Keywords:** calcium oxalate, dietary oxalate, kidney stones, metabolism, nephrolithiasis, oxalate, oxalate synthesis, urolithiasis

## Abstract

Kidney stone disease is increasing in prevalence, and the most common stone composition is calcium oxalate. Dietary oxalate intake and endogenous production of oxalate are important in the pathophysiology of calcium oxalate stone disease. The impact of dietary oxalate intake on urinary oxalate excretion and kidney stone disease risk has been assessed through large cohort studies as well as smaller studies with dietary control. Net gastrointestinal oxalate absorption influences urinary oxalate excretion. Oxalate-degrading bacteria in the gut microbiome, especially *Oxalobacter formigenes,* may mitigate stone risk through reducing net oxalate absorption. Ascorbic acid (vitamin C) is the main dietary precursor for endogenous production of oxalate with several other compounds playing a lesser role. Renal handling of oxalate and, potentially, renal synthesis of oxalate may contribute to stone formation. In this review, we discuss dietary oxalate and precursors of oxalate, their pertinent physiology in humans, and what is known about their role in kidney stone disease.

## 1. Introduction

Kidney stone disease affects approximately 10% of the population [1] and calcium oxalate (CaOx) is the most common stone composition [2]. Supersaturation of urine with CaOx markedly increases the risk of stone formation [3], and supersaturation is the driving force behind CaOx crystal precipitation [4]. The contribution of urinary oxalate to supersaturation of CaOx is significant. For example, in one study, the supersaturation of CaOx was up to 23 times more sensitive to changes in urinary oxalate compared to changes in urinary calcium [5]. In a subsequent study, urinary calcium and urinary oxalate were found to contribute equally to supersaturation of CaOx [6]. Urinary oxalate is derived from dietary oxalate intake and endogenous oxalate synthesis [7]. The diet-derived portion depends primarily on three factors: the amount of oxalate consumed, the amount of calcium and other divalent cations consumed, and oxalate handling by the intestine. This is further complicated by the degradation of some of the ingested oxalate by intestinal bacteria [8] and differences in oxalate absorption in different regions of the intestinal tract [9]. Endogenous oxalate production occurs primarily in the liver and is influenced by dietary intake of precursors, notably ascorbic acid (AA) [10]. Subsequent renal handling [11] and, perhaps, endogenous synthesis by the kidney are final determinants of the urinary oxalate pool. In this paper, we review sources of dietary oxalate and oxalate precursors, their impact on the urinary oxalate pool, and their influence on kidney stone risk.

## 2. Dietary Oxalate Intake and Urinary Oxalate Excretion

Several methods have been utilized to evaluate dietary oxalate and its impact on urinary oxalate and kidney stone disease. These include food frequency questionnaires (FFQs) from large epidemiologic studies. While a number of these studies are central to this review (Table 1A), FFQs have particular limitations pertaining to dietary oxalate. While smaller in scale, studies involving subjects on controlled diets offer strict regulation of factors influencing urinary oxalate excretion (Table 1B).

### 2.1. Quantifying Oxalate Consumption

An estimate of the amount of oxalate consumed in large populations has been performed primarily by the use of FFQs. This tool was crucial in identifying the critical role of dietary calcium in CaOx stone formation [32]. The main mechanism for this effect was proposed to be the binding of calcium to oxalate limiting intestinal oxalate absorption [33]. Through a similar approach, however, the relationship between dietary oxalate and kidney stone disease was less pronounced [12]. Furthermore, the difference in urinary oxalate between the highest and lowest quartiles of oxalate consumption based on an FFQ was small, 1.7 mg/day [13]. There are several reasons for these inconsistencies:

(1) The daily intake of oxalate on a molar basis is much less than calcium, approximately one-tenth (2.5 mmol vs. 25 mmol);
(2) The amount of bound oxalate ingested is higher than bound calcium;
(3) The amount of oxalate ingested can be difficult to determine due to the variability of oxalate in plants and plant-based foods. These differences may be due to growth conditions, genetic divergence and analytical variability in oxalate analyses [34];
(4) FFQs are subject to errors and when targeting a single nutrient, they should be validated by comparison with another technique such as a weighed 3–4-day food record [35].

Due to the plurality of these influencing factors, it would appear that validating the accuracy of FFQs in determining oxalate consumption could be a fruitless, if not impossible task. Studies utilizing controlled dietary conditions overcome many of the limitations of FFQs with respect to oxalate intake.

### 2.2. Relationship between Dietary Oxalate and Urinary Oxalate

Several studies have been performed in healthy human subjects that have addressed the relationship between dietary oxalate to its urinary excretion using diets tightly controlled in their nutrient content. In one study, the mean contribution of dietary oxalate to urinary oxalate ranged from 25% (10 mg dietary oxalate per day) to 42% (250 mg dietary oxalate per day); when the calcium content of the 250 mg oxalate/day diet was decreased from 1002 mg to 391 mg, the mean contribution increased to 53% [7]. Further analysis demonstrated a linear increase in urinary oxalate excretion with respect to dietary oxalate intake over a range of 50–750 mg daily intake under defined dietary conditions including calcium intake of 1000 mg/day [36]. It is important to note that dietary oxalate intake on self-selected diets can vary by more than 200 mg/day [37], producing substantial variation in oxalate excretion without dietary control. We have previously advocated that reducing or eliminating oxalate-rich foods in the diet, when appropriate, should be beneficial in reducing the contribution of diet to urinary oxalate excretion [38].

## 3. Dietary Oxalate and the Gut

Gastrointestinal oxalate absorption is influenced by absorptive and secretory fluxes of this dicarboxylic acid. The influence of oxalate-degrading organisms in the fecal microbiome including *Oxalobacter formigenes* on these processes is an area of active research.

### 3.1. Gut Absorption of Dietary Oxalate

Oxalate is absorbed along the intestinal tract via transcellular anion transporters, notably the solute-linked carrier (SLC)-26 family, as well as paracellular fluxes through tight junctions [9,39]. The amount of ingested oxalate that is absorbed by the gut appears to be between 5 and 15%, depending on intake of calcium, magnesium, and fiber [15]. Hyperabsorption of oxalate has been associated with surgical resection of intestinal segments, including bypass surgery for weight loss, and malabsorptive intestinal diseases. This secondary hyperoxaluria, known as enteric hyperoxaluria (reviewed in [40]), has been attributed to the increased amount of soluble oxalate available for absorption as a consequence of free fat malabsorption resulting in sequestration and saponification of calcium, as well as the presence of increased amounts of bile acids, again due to malabsorption, which is thought to augment gastrointestinal oxalate uptake.

### 3.2. Gut Microbial Oxalate Degradation

The mammalian body does not possess enzymes capable of metabolizing oxalate. However, multiple bacterial species in the gastrointestinal tract have the ability to degrade oxalate, and thus it is hypothesized these gut microbes play an important role in reducing the risk of CaOx stone disease. There are two major groups of oxalate-degrading bacteria in the gastrointestinal tract. There are the “generalist oxalotrophs”, which do not depend entirely on oxalate as an energy source, and the “specialist oxalotrophs”, which use oxalate as their sole or major carbon and energy source [41]. To date, only one specialist oxalotroph has been discovered in the mammalian gut, the obligate anaerobic bacterium *O. formigenes* [42,43]. Most of the research has centered on this bacterium [44,45,46,47,48,49], with only a few studies addressing the role of the general oxalotrophs on stone disease [50,51].

A few studies have examined the impact of probiotic preparations designed to degrade gastrointestinal oxalate with the intent of attenuating urinary oxalate excretion, including those containing *O. formigenes*, *Lactobacillus*, and/or *Bifidobacterium spp.* [23,52,53,54]. Although these trials did not show a significant effect on urinary oxalate excretion, it is not clear whether the ingested bacteria in these trials maintained viability. Enzymatic degradation of oxalate throughout the gastrointestinal tract using an oral preparation of oxalate decarboxylase is a more recent potential treatment strategy [55]. A phase 2, open-label trial found that dosing with oxalate decarboxylase significantly reduced 24-h urinary oxalate excretion in participants with idiopathic (−10.2 mg) and enteric hyperoxaluria (−22.0 mg) [56]. The relative role of generalist oxalotrophs and *O. formigenes* on gut oxalate degradation has only been examined in one study with healthy non-stone formers [25]. This study utilized diets controlled in nutrients, including oxalate, and demonstrated that the oxalate degrading capacity of the microbiome of individuals not colonized with *O. formigenes* is negligible at low dietary oxalate intake and increases with ingestion of higher levels of dietary oxalate. This is consistent with early dietary oxalate feeding studies in ruminants [57]. This study also found that individuals colonized with *O. formigenes* excreted significantly lower levels of fecal oxalate compared with those individuals not colonized with *O. formigenes*, highlighting the importance of a specialist oxalotroph on overall gut oxalate degradation. For example, at a moderately high intake of dietary oxalate (250 mg per day), 80% of the dietary oxalate ingested was recovered in feces in individuals not colonized with *O. formigenes*, whereas only 30% was recovered in feces in individuals colonized with *O. formigenes*.

Similar controlled dietary studies need to be performed in stone formers to determine the relative role of generalist oxalotrophs and *O. formigenes* on gut oxalate degradation, and how colonization of the gut with these microbes impacts urinary oxalate excretion and risk of stone formation. Future studies utilizing state of the art sequencing technologies [58] could lead to the identification of networks of microbes that play an important role in enhancing oxalate degradation in the human gastrointestinal tract. Furthermore, as oral antibiotic use and antibiotic exposure at a young age have been associated with an increased risk for stone formation [59], future research should also focus on determining the mechanisms by which antibiotic exposure increases the risk of stone disease, including the impact of antibiotic treatment on microbial oxalate degradation.

## 4. Precursors of Endogenous Oxalate Production

**Figure 1:**
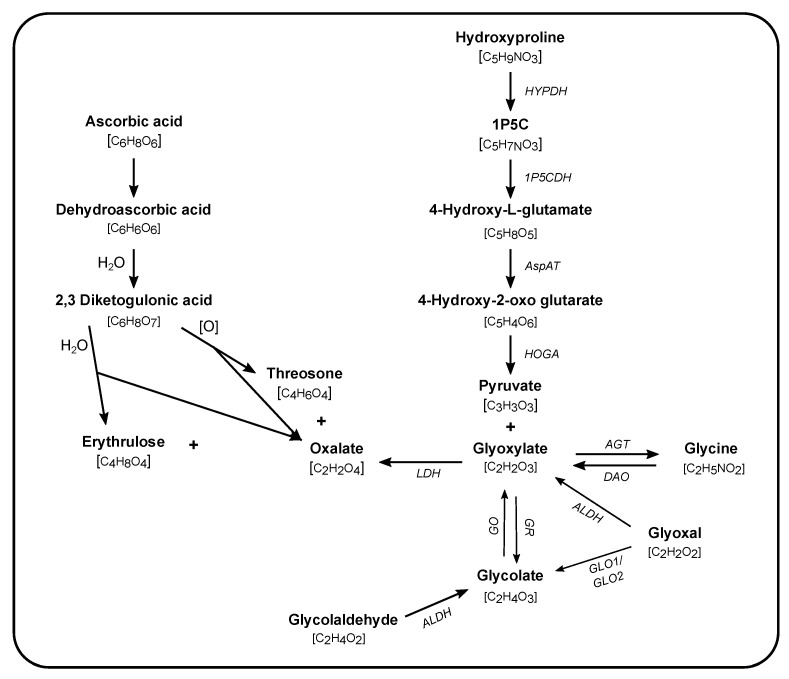
Pathways of endogenous oxalate synthesis. Synthesis of oxalate occurs via enzymatic (names in italics) and non-enzymatic reactions taking place in different subcellular compartments (cytosol, mitochondria, and peroxisome). The molecular formula is given in brackets underneath each compound. Glycolaldehyde is a product of fructose and xylitol metabolism. 1P5C: 1-pyrroline-3-hydroxy-5-carboxylic acid, LDH: lactate dehydrogenase, GO: glycolate oxidase, GR: glyoxylate reductase, AGT: alanine:glyoxylate aminotransferase, DAO: D-amino oxidase, ALDH: aldehyde dehydrogenase, GLO: glyoxalase, HOGA: 4-hydroxy-2-oxoglutarate aldolase, AspAT: aspartate aminotransferase, 1P5CDH: 1P5C dehydrogenase, HYPDH: 4-hydroxyproline dehydrogenase.

The initial insights into endogenous oxalate synthesis were generated over 60 years ago by the study of the rare hereditary kidney stone/nephrocalcinosis generating diseases, primary hyperoxalurias (PH). Studies using ^14^C- and ^13^C-labeled compounds in rats and in healthy volunteers and patients with PH were instrumental in determining the importance of the liver in oxalate production and in identifying precursors to oxalate synthesis (Figure 1). The central role of the liver in oxalate synthesis was demonstrated by studies performed in isolated rat liver and hepatectomized rats [60,61]. The contribution of other tissues, such as the kidney, have yet to be fully elucidated. In the liver, glyoxylate has been identified as a direct precursor to oxalate, and lactate dehydrogenase as the key enzyme in oxidizing glyoxylate to oxalate [62,63,64,65]. Further supporting the central role of the liver in glyoxylate to oxalate metabolism, the enzyme deficiencies in PH are involved in glyoxylate metabolism and are highly or even entirely expressed in the liver [66,67,68,69,70]. Thus, the search for precursors to oxalate synthesis has been driven by pathways involving glyoxylate metabolism [71,72]. A notable exception is AA, which has long been known to be a non-enzymatic source of oxalate synthesis [73,74].

### 4.1. Amino Acids and Proteins

Among the oxalate precursors that have been studied, a number of amino acids have been proposed (glycine, serine, tyrosine, tryptophan, phenylalanine, and hydroxyproline), but only a few have been evaluated and validated in humans, fewer still with dietary control [60,75,76,77,78]. The role of glycine in oxalate synthesis was initially thought to be a major one with contribution to oxalate synthesis estimated as high as 40% in older studies [79,80,81]. However, a study of 6 healthy volunteers using improved analytical method including dietary control, and primed, constant infusions of ^13^C-glycine demonstrated that glycine metabolism contributed <5% to urinary oxalate [26]. Hydroxyproline, a product of collagen breakdown, when orally ingested in the form of gelatin, showed conversion to oxalate in healthy volunteers [19]. This study prompted a more quantitative examination of hydroxyproline catabolism to oxalate using primed, constant infusions of ^15^N, ^13^C_5_-hydroxyproline in healthy volunteers and subjects with PH, on controlled diets [30]. This study showed that the metabolism of hydroxyproline contributed to at least 15% of endogenous oxalate synthesis in healthy subjects and played a greater role in patients with PH, notably PH type 2 and type 3.

### 4.2. Other Sources of Glyoxylate

Glycolate, a precursor to glyoxylate, is a major source of oxalate synthesis in PH type 1. Its metabolism to glyoxylate has been established by numerous studies in humans and rodents for over 60 years [60,61,63,71,82]. Inhibition of glycolate oxidation to glyoxylate by RNA interference or CRISPR/Cas9 targeting of glycolate oxidase (GO) has shown effectiveness in reducing urinary oxalate excretion in mice deficient in alanine:glyoxylate aminotransferase (AGT), a model of PH type 1 [83,84,85]. The central role of GO to enhanced oxalate synthesis in PH type 1 stems from the deficiency in AGT; however, in healthy volunteers with normal AGT activity, the role of glycolate as an oxalate precursor is unknown. Glyoxal, a 2-carbon reactive dialdehyde, is produced through a number of oxidative and metabolic reactions [86] and can be enzymatically converted to glyoxylate [87] and glycolate [88]. In a study utilizing human erythrocytes, glyoxal was shown to be preferably converted to glycolate with 1% ultimately converted to oxalate via glyoxylate [89]. The formation of oxalate was increased when intracellular glutathione was depleted, highlighting a potential role of oxidative stress in endogenous oxalate synthesis through this pathway. Glyoxal is found in a vast number of food items and beverages, such as bread, cookies, yogurt, sardine oil, coffee, tea, beer and wine, and is a food processing contaminant [90,91,92,93,94,95]; however, nothing is known about the intestinal absorption of these dietary sources of glyoxal. The reactivity of glyoxal in vivo also makes it a difficult target to study and its role in endogenous oxalate synthesis remains unclear.

### 4.3. Fructose, Glucose and Pentose Sugars

Epidemiological evidence of the association between fructose intake and kidney stone formation [96,97] supports the hypothesis that fructose consumption could lead to oxalate synthesis, directly or indirectly through serine metabolism [77]. However, a controlled diet study in healthy volunteers fed with increasing fructose (4–21% of calories) failed to demonstrate a change in urinary oxalate excretion [22]. Furthermore, in vitro experiments in liver cells did not show any conversion of ^13^C-labeled sugars (fructose and glucose) to oxalate [22]. The reported association of fructose intake and kidney stone disease may be explained by confounding factors such as obesity and insulin resistance [98,99]. The pentose pathway and xylulose has been proposed as a source of oxalate synthesis, via glycoladehyde and glycolate synthesis [77]. Although there have been case reports of oxalosis following xylitol intake, in vitro experiments [100] do not support xylitol as an important source of oxalate, and thus it is still unclear whether the intake of xylitol is a risk factor for increased urinary oxalate excretion.

### 4.4. Ascorbic Acid

The relationship between AA and oxalate synthesis has been known since the mid-20th century [73]. The formation of oxalate is the result of the non-enzymatic breakdown of AA into 2,3-diketogulonic acid (DKG) (Figure 1). DKG can be split into threosone or erythrulose, accompanied by oxalate in both cases [10,74]. The conversion is pH and temperature sensitive, a factor that may impact oxalate measurement in some biological samples rich in AA. Studies by Atkins et al. and Baker et al. indicate that AA may be the largest contributor to endogenous oxalate synthesis [81,101], contributing approximately 40% to urinary oxalate excretion. The methods used at that time had limitations and thus studies readdressing the role of AA in endogenous oxalate synthesis are needed. The association between AA intake and the risk of kidney stone risk has been shown in several large cohort studies, although lack of dietary control, short duration of the studies and potential sample handling issues may have influenced the results [13,102]. AA intake has also been reported to promote oxalate excretion to a greater degree in kidney stone formers [13]. Though one study performed under conditions of dietary control found that both kidney stone formers and healthy controls had increased urinary oxalate attributed to oral AA supplementation [17]. Several case reports have also indicated that individuals with compromised renal function can develop oxalate nephropathy following large oral doses or intravenous infusions of AA [103,104,105]. Despite the importance of AA to oxalate synthesis in both healthy subjects and kidney stone patients, there remain gaps in our knowledge of the mechanisms involved. Mitochondria have been regarded as a major site of intracellular AA recycling and act as a major component of endogenous antioxidant defense [106]. It is possible that as AA acts as an antioxidant in the mitochondria in which oxalate is produced. Investigating the importance of this pathway in oxalate synthesis is warranted and may identify novel approaches to reducing oxalate synthesis.

## 5. Oxalate and the Kidney

Oxalate is an end product of metabolism in humans. In healthy subjects, approximately 95% of the oxalate absorbed by the gut and produced endogenously is excreted in urine [107]. Oxalate is delivered to the nephron through glomerular filtration and secretion [108,109]. While oxalate reabsorption has been demonstrated in the rat proximal tubule [110], this has not been characterized in humans. Oral loading of a single dose of sodium oxalate in healthy volunteers on controlled diets showed a rapid absorption and net renal secretion, with transient elevations in both urinary and plasma oxalate [11]. A later study evaluating oxalate excretion following oral oxalate loads demonstrated no differences in urinary oxalate excretion or the ratio of oxalate to creatinine clearance between normal subjects and kidney stone formers [20]. However, Bergsland et al. found that patients with idiopathic hypercalciuria or stone formers who have had bariatric surgery had significantly more rapid increases in urinary oxalate compared to normal controls while on controlled diets, and that tubular secretion of oxalate was a key mediator, potentially to maintain tight regulation of plasma oxalate [24]. In addition to its role in excreting oxalate, there is some evidence that the kidney might play a role in oxalate synthesis. Both glyoxylate reductase (GR) and 4-hydroxyproline dehydrogenase (HYPDH) enzymes are highly expressed in the kidney. In rats, the proximal tubule has been shown to be the segment of the nephron where these enzymes are highly expressed [111,112,113,114]. Experiments by Farinelli and Richardson in the hepatectomized rat have suggested that some metabolism of glycolate to oxalate might occur in the kidney via unknown mechanisms [61]. A study in the GR-deficient mouse, a model of PH2, has shown that dietary hydroxyproline feeding rapidly induces CaOx nephrocalcinosis due to increased renal oxalate synthesis [115]. In light of these findings, future studies should examine the role of renal oxalate synthesis and renal oxalate handling in urinary oxalate excretion.

## 6. Conclusions

Intake of dietary oxalate and dietary oxalate precursors are key determinants of urinary oxalate excretion and CaOx stone disease. Quantification of dietary oxalate intake in large populations via FFQs has significant limitations which can be overcome through studies with strict dietary control. Through such studies, it has been determined that dietary oxalate intake on average accounts for ~50% of urinary oxalate excretion in normal healthy subjects. The role of oxalate-degrading gut microbes in CaOx kidney stone disease is still unclear and requires further investigation using controlled diets. AA has been identified as the major dietary oxalate precursor while other putative precursors seem to have a lesser role. However, a limitation of the majority of these studies is they have only been conducted in healthy volunteers. Controlled dietary studies in patients with stone disease to examine the role of dietary oxalate, oxalate precursors, and both renal and gastrointestinal handling are needed.

## Figures and Tables

**Table 1:** Selected studies with methods and clinical outcomes relevant to oxalate assessed through (**A**) food frequency questionnaires in large cohorts or (**B**) dietary control in small cohorts.

Ref.	Year	Number of Participants	Subject Population	Variables or Interventions Studied	Clinical Outcome	Findings/Conclusions
**A.** **Large cohort studies utilizing food frequency questionnaires**						
[12]	2007	240,681	Female registered nurses and male health professionals	Dietary oxalate	Kidney stone risk	Oxalate intake was not strongly associated with stone risk
[13]	2008	3348	Female registered nurses and male health professionals	Dietary oxalate, ascorbic acid supplementation	Urinary oxalate	The impact of dietary oxalate on urinary oxalate was small; ascorbic acid supplementation significantly increased urinary oxalate
[14]	2016	197,271	Female registered nurses and male health professionals	Dietary and supplemental ascorbic acid intake	Kidney stone risk	Total and supplemental ascorbic acid intakes were associated with increased stone risk for men, but not women
**B.** **Small cohort studies utilizing controlled diets**						
[15]	1995	2	Normal healthy subjects	Dietary oxalate (oxalate-free formula)	Urinary oxalate	Intestinal absorption of oxalate contributes significantly to urinary oxalate
[7]	2001	12	Normal healthy subjects	Dietary oxalate (10–250 mg/day), dietary calcium (391–1002 mg/day)	Urinary oxalate	Dietary oxalate accounts for up to 50% of urinary oxalate, depending on calcium intake
[16]	2001	22	Normal healthy subjects	High-oxalate, low-calcium diet	Urinary oxalate	White participants had significantly higher urinary oxalate compared to black participants
[17]	2003	24	Kidney stone formers (*n* = 12) and normal healthy subjects (*n* = 12)	Oral ascorbic acid supplementation (2 g/d)	Urinary oxalate	Ascorbic acid supplementation significantly increased urinary oxalate in both stone formers and normal subjects
[18]	2004	48	Kidney stone formers (*n* = 29) and normal healthy subjects (*n* = 19)	Oral load of ^13^C-oxalate and oral ascorbic acid supplementation (2 g/d)	Urinary oxalate	Stone formers had higher oxalate absorption. Ascorbic acid supplementation increased urinary oxalate
[11]	2005	6	Normal healthy subjects	Oral oxalate loads (0–8 mmole)	Urinary oxalate	Oxalate is rapidly filtered and secreted by the kidney following an oral load in a dose-dependent fashion
[19]	2006	10	Normal healthy subjects	Oral hydroxyproline in the form of gelatin	Urinary oxalate	Hydroxyproline metabolism contributes 5–20% of urinary oxalate derived from endogenous synthesis
[20]	2007	12	Kidney stone formers (*n* = 6) and normal healthy subjects (*n* = 6)	Oral oxalate loads (0–8 mmole)	Urinary oxalate	No significant difference in urinary oxalate excretion between stone formers and normal subjects
[21]	2009	11	Normal healthy subjects	Dietary protein (0.6–1.8 g/kg body weight)	Urinary oxalate	Increased protein intake is not associated with increased urinary oxalate excretion
[22]	2010	7	Normal healthy subjects	Dietary fructose (4–21% of calories)	Urinary oxalate	No change in urinary oxalate was observed with increasing fructose intake
[23]	2010	40	Hyperoxaluric calcium oxalate stone formers	Probiotic preparations	Urinary oxalate	The probiotics tested did not reduce urinary oxalate excretion under conditions of dietary oxalate restriction
[24]	2011	29	Idiopathic hypercalciurics (*n* = 19), bariatric stone formers (*n* = 2), normal healthy subjects (*n* = 8)	Diet containing 92 mg oxalate	Urinary oxalate	Urinary oxalate excretion and renal oxalate secretion were significantly higher in patients compared to healthy subjects
[25]	2011	22	Normal healthy subjects	Colonization with *Oxalobacter formigenes*	Urinary oxalate	Colonization with *O. formigenes* decreases urinary oxalate excretion under conditions of low calcium and moderate calcium intake
[26]	2011	6	Normal healthy subjects	Infusions of ^13^C-glycine and ^13^C-phenylalanine	Urinary oxalate	Glycine and phenylalaine make minor contributions to endogenous oxalate production and urinary oxalate excretion
[27]	2012	10	Normal healthy subjects	Dietary calcium/oxalate balance	Urinary oxalate	Ingesting large quantities of oxalate likely does not impact calcium oxalate stone risk if recommended daily dietary calcium is consumed
[28]	2012	9	Bariatric stone formers	Normal calcium, low oxalate diet	Urinary oxalate	No significant change in urinary oxalate on controlled diet compared to self-selected diet
[29]	2014	15	Normal healthy subjects	Fish oil supplementation	Urinary oxalate	Fish oil supplementation does not decrease urinary oxalate during periods of extremely low dietary oxalate
[30]	2018	28	Primary hyperoxaluria types 1/2/3 (*n* = 19), normal healthy subjects (*n* = 9)	Infusion of ^15^N, ^13^C-hydroxyproline	Urinary oxalate	Hydroxyproline contributes 15% to endogenous oxalate production in healthy subjects, with greater contribution in all primary hyperoxalurias
[31]	2020	14	Normal healthy subjects	Single oral oxalate load (8 mmole)	Urinary oxalate	Urinary oxalate increases 5 h after consumption of a blended preparation of fruits and vegetables

## Data Availability

No new data were created or analyzed in this study. Data sharing is not applicable to this article.
